# Editorial: Evolution of crop genomes and epigenomes, volume II

**DOI:** 10.3389/fpls.2025.1623554

**Published:** 2025-06-16

**Authors:** Hai Du, Zhe Liang, Andrés J. Cortés

**Affiliations:** ^1^ College of Agronomy and Biotechnology, Chongqing Engineering Research Center for Rapeseed, Southwest University, Chongqing, China; ^2^ Biotechnology Research Institute, Chinese Academy of Agricultural Sciences, Beijing, China; ^3^ Corporación Colombiana de Investigación Agropecuaria (AGROSAVIA) – C.I. La Selva, Rionegro, Colombia; ^4^ Facultad de Ciencias Agrarias-Departamento de Ciencias Forestales, Universidad Nacional de Colombia – Sede Medellín, Medellín, Colombia

**Keywords:** multi-omics, genomics, epigenomics, epitranscriptomics, transcriptomics, metabolomics, gene evolution, pathway evolution

Darwin’s theory (1859) explained natural phenotypic variation and selection but lacked a mechanism for inheritance (Darwin, 1859), later addressed by Mendel’s rediscovery and the Modern Synthesis (Huxley, 1943). Advances in genetics expanded evolutionary theory to genomic and epigenomic levels, revealing polygenic/omnigenic architectures (Barghi et al., 2020; Boyle et al., 2017) and transgenerational epigenetic inheritance (Boskovic and Rando, 2018). Domestication, Darwin’s proof-of-concept for selection (Darwin, 1868), now aids genomic/epigenomic research in crops (Alam and Purugganan, 2024). Multi-omics studies (Meyer and Purugganan, 2013; Gutaker and Purugganan, 2023) elucidate crop adaptation, domestication, and agronomic gene discovery (Joly-Lopez et al., 2016). Advances in sequencing, machine learning, and bioinformatics (Cortés and López-Hernández, 2021) have accelerated crop genome research (Cortés et al., 2023), revealing how domestication reshaped genomes/epigenomes (Purugganan, 2022).

A deeper understanding of these evolutionary constrains and changes is crucial for developing superior and sustainable crop varieties with enhanced yield, nutritional value, and stress resilience. This Research Topic explores crop genome/epigenome evolution through multi-omics analyses, compiling discoveries across gene families, pathways, and diverse species. This Research Topic comprises five original research articles focusing on the above research areas, viewed 7,387 times by the time of this Editorial. These works enable readers to *(i)* quantify the scale of divergence and conservation of genomes and epigenomes during crop evolution, *(ii)* reconstruct the evolutionary history of target gene families and pathways, *(iii)* expand the paradigm of molecular evolution to acknowledge variable gene expression, gene regulatory and metabolomic profiles into what nowadays can be recognized as multi-omic evolution, *(iv)* identify patters and causal relationships between genome size, genome duplication/polyploidy, and the occurrence of key evolutionary innovations, and ultimately *(v)* interpret the metabolomic/phenotypic consequences of genome/epigenome evolution. All insights leverage large-scale multi-omics data with biotech/agricultural applications.

## Multi-omic evolution – nothing makes sense in molecular evolution except in the light of the multi-omics spectrum

First, Li et al. adventured into the evolution of mitochondrial genomes, a long-standing question in molecular genetics, within the *Saccharum* complex. The researchers assembled and compared the graph-based mitochondrial genomes of four *Saccharum* species related to sugarcane (*i.e.*, *Tripidium arundinaceum*, *Erianthus rockii*, *Miscanthus sinensis*, and *Narenga porphyrocoma*) using Illumina and PacBio HiFi data. Comparative genomics analyses revealed significant structural variations and phylogenetic relationships. For instance, the authors found that the mitogenomes exhibited complex, graph-based structures with multiple junctions. They identified a total of 51 unique genes in the mitogenomes, including 32 protein-coding genes (PCGs), 16 tRNA genes, and 3 rRNA genes. Authors also traced the sequences transferred from the chloroplast to the mitogenome, with *M. sinensis* showing the highest transfer length and proportion. Based on the phylogenetic analysis of 13 conserved mitochondrial PCGs, the authors concluded that *N. porphyrocoma* was the closest relative to *Saccharum*. They also unveiled the extensive genomic rearrangements among the mitogenomes, and highlighted the dynamic nature of mitochondrial genome evolution, including gene duplication and loss, with the ATP synthase and cytochrome *c* synthesis genes being the most conserved likely due to puryfing selection. The significance of this study lies in its contribution to comparative genomic studies and the enrichment of genomic resources relevant to sugarcane breeding. The identification of structural variations and phylogenetic insights directly address the need for more comparative studies and mechanistic understanding of organelle evolution. Ultimately, this study enriches the mitochondrial genomic resources for Saccharinae and provides new insights into the evolution of mitogenomes at the family and genus levels.

On a more gene-target spectrum, Yang et al. conducted a genome-wide study of the EIN3/EIL gene family in broomcorn millet (*Panicum miliaceum* L.). Phylogenetic analysis and expression profiling highlighted diverse spatiotemporal and stress-related expression patterns, not to mention variation in protein structure and *cis*-regulatory elements in the promoter region. Specifically, the authors identified 15 EIN3/EIL genes in *P. miliaceum*, which can be classified into four groups based on the conserved motif composition and gene structure features. They verified that genome-wide duplication events mainly contributed to the gene expansion, and all duplicated genes undergone purifying selection during their recent evolution. This enabled the expansion of biological functions within the gene family, impacting different growth and developmental stages in *P. miliaceum* as well as response to abiotic stresses, include cold, drought and salt stresses. The work provides an integrative analysis of a gene family with widespread phenotypic effects in a polyploid crop, offering valuable insights into the effects of polyploidization on gene family evolution and function.

Similarly, Liu et al. presented a systematic identification and evolutionary analysis of the glucosinolate (GSL) pathway genes in 14 representative plant genomes, with focus on the evolution and comparative transcriptome analysis in the oil crop *Brassica napus* L. The authors identified a total of 1280 genes in the GSL pathway from across 14 species. They further demonstrated that these genes are specifically distributed in Brassicaceae and are extensively expanded in *B. napus*. The analyses revealed that whole-genome duplication events contributed to the large gene expansion of the GSL pathway in *B. napus*. Meanwhile, this study built a comprehensive RNA-seq dataset of a high- (ZY821) and a low-GSL-content (ZS11) *B. napus* cultivar, which enabled studying differences in the expression profiles across tissues/organs at different stages. Based on this RNA-seq data, authors identified 65 differential expressed genes (DEGs) that may determine the differences in GSL content between ZY821 and ZS11. The study provides an inclusive dataset of GSL pathway genes, enhancing our understanding of pathway evolution and providing valuable resources for *B. napus* improvement through molecular breeding (Cortés and Du, 2023).

Meanwhile, Liu et al. explored the functional diversity of cytochrome P450 enzymes (CYP96Ts) involved in Amaryllidaceae alkaloid biosynthesis. The authors generated a full-length transcriptome of *Lycoris aurea* by PacBio single-molecule real-time (SMRT) sequencing to trace the function of *L. aurea* CYP96T1-like cytochrome P450 in alkaloid biosynthesis. They obtained a total of 52,338 unigenes in this species based on the transcriptome data, and identified five unigenes related to oxidative-coupling cytochrome P450 based on diverse *L. aurea* cDNA library analyses, co-expression analysis and RT-PCR assay. Four candidate genes (*LauCYP96T1*, *LauCYP96T1-like-1*, *LauCYP96T1-like-2*, and *LauCYP96T1-like-3*) were cloned, to later be used for functional characteristic analysis (*i.e.*, subcellular localization analysis, expression and *in vitro* enzymatic reaction assay, and structural homology modeling analysis), revealing inverted regioselectivity for oxidative coupling of 4’-O-methylnorbelladine. The authors elucidated that these four *CYP96T* homologs catalyzed *para-para*´ and *para-ortho*´ oxidative coupling in Amaryllidaceae alkaloids biosynthesis. This research brings insights into the functional diversity and pleiotropy of CYP96T enzymes, highlighting the need for deeper mechanistic understanding of specialized pathways.

Finally, on a more downstream level, Lin et al. delved the intricate relationship between gene expression and metabolic profiles during sweet potato tuber development. By employing RNA sequencing and metabolomics, the authors investigated the gene expression and metabolic profiles during the tuber development (70, 100, and 130 days). They identified 16,303 DEGs and 1,566 differentially regulated metabolites (DRMs). DEGs and DRMs were significantly enriched in the pathways related to starch and sucrose metabolism, and flavonoid biosynthesis. The authors further pinpointed 14 candidate genes related to starch, carotenoids and anthocyanins contents in sweet potato tubers, *i.e*., chalcone isomerase (*CHI*) gene in flavonoid biosynthesis, and UDP-glucose pyrophosphorylase 2 (*UGP2*) and glycogen synthase (*glgA*) genes in starch biosynthesis. The landmark of this work consists in providing a detailed molecular-level understanding of the regulatory mechanisms governing tuber development by carrying out multi-omic data integration.

## Expanding the frontiers of crop genome and epigenome research

The findings highlighted in this Research Topic lay the foundation for innovative research in the nascent field of multi-omic evolution. Among the several key areas that warrant further exploration, expanding the species scope is perhaps the most imperative. The studies presented here focus on a select group of crop species, yet future research should expand the species spectrum to encompass a broader representation of crops, particularly those with unique evolutionary histories (e.g., Cortés et al., 2018), orphan research (Hu et al., 2025), or paramount importance for the food security (e.g., López-Hernández et al., 2023), nutrition (Wu et al., 2020, 2024), sustainability (Benitez-Alfonso et al., 2023) and self-sufficiency (Scherer et al., 2020; Varshney et al., 2021b) targets. This will enlighten more generalizable patterns of crop genome and epigenome evolution.

Second, but not less important, a more prominent integration of epigenetic regulation is desirable. Epigenetic modifications play a crucial role in shaping gene expression (Chinnusamy and Zhu, 2009) and phenotypic plasticity (Kristensen et al., 2020; Fox et al., 2019), yet are often disregarded by studies focusing on the latter paradigms (Bossdorf et al., 2008). Oncoming research should aim integrating epigenomic data (*e.g.*, DNA methylation, histone modifications) with genomic and transcriptomic data to build a more comprehensive understanding of the evolutionary constrains shaping crop genomes and epigenomes, and their phenotypic consequences.

On a third note, interpreting evolution across the multi-omics continuum requires more advanced modeling techniques. The development of sophisticated computational models capable of predicting the multi-dimensional downstream consequences of genomic and epigenomic variations on crop traits and metabolomes is crucial. These models should be capable to simultaneously integrate multiple data types and incorporate information on gene regulatory networks and their environmental interactions. The current machine learning boom (Varshney, 2021) promises assisting in these matters (Libbrecht and Noble, 2015; Schrider and Kern, 2018).

## Perspectives

While substantial progress has been made in recognizing major trends and causes during crop genome evolution, as illustrated by this Research Topic, critical knowledge gaps remain. A more comprehensive understanding requires the integration of diverse data types – genomic, transcriptomic, epigenomic, and metabolomic – to build a holistic picture of evolutionary changes at intricate omic levels (Barrera-Redondo et al., 2020). Meanwhile, further comparative studies across closely related species are essential to disentangle common evolutionary trajectories (Wolf and Ellegren, 2017) from unique adaptations driven by specific environmental pressures or selective breeding (Feng et al., 2024). Despite recent efforts in these last two fronts, the mechanistic comprehension of coupled genomic and epigenomic changes, and their downstream consequences, are still in its infancy. Therefore, identifying the key genes and regulatory pathways involved, as well as their environmental context (Cortés et al., 2022; Lasky et al., 2023), is crucial for a complete understating of these evolutionary processes. Equally important, translating fundamental mechanistic knowledge into practical applications for crop improvement is critical, especially due to a limited adoption of innovation by farmers (Kholova et al., 2024). This will require strategies that harness multi-omic evolutionary novelty to speed breeding programs aiming at the design of crops with superior resilience to biotic and abiotic stresses (Varshney et al., 2021a; Cortés, 2024), without disregarding their market value and the farmers’ preferences (Peláez et al., 2022).

Visualizing these novel trends in the field of multi-omic evolution will in turn impact other transgressive technologies, such as gene editing. Target genes and regulatory elements identified as part of these studies can be manipulated by unprecedented genome editing tools such as CRISPR/Cas9 (Doudna and Charpentier, 2014). For instance, this approach is now allowing precise editions to test gene functionality in crop backgrounds, and eventually leverage them for fast-forward improvement (Dort et al., 2020), while recognizing intrinsic evolutionary trade-offs (Denison, 2016) and genomic constrains (Ellegren and Galtier, 2016).
